# A real-world assessment of mycophenolate mofetil for remission induction in eosinophilic granulomatosis with polyangiitis

**DOI:** 10.1007/s00296-021-04961-w

**Published:** 2021-08-04

**Authors:** Mariana Philobos, Amy Perkins, Maira Karabayas, Paula Dospinescu, Nick Fluck, Dana Kidder, Fiona A. Chapman, Neeraj Dhaun, Neil Basu

**Affiliations:** 1 Rheumatology Unit, NHS Grampian, Aberdeen, UK; 2 Renal Unit, Aberdeen Royal Infirmary, NHS Grampian, Aberdeen, UK; 3 Renal Unit, Edinburgh Royal Infirmary, NHS Lothian, Edinburgh, UK; 4 Institute of Infection, Immunity and Inflammation, University of Glasgow, Glasgow, UK

**Keywords:** Mycophenolate, Vasculitis, Egpa

## Abstract

Eosinophilic granulomatosis with polyangiitis (EGPA) is a form of ANCA-associated vasculitis (AAV). Clinical trials demonstrating the efficacy of mycophenolate mofetil (MMF) for remission induction in AAV excluded patients with EGPA. Despite this, MMF is commonly used in these patients. The objective of this study was to evaluate, for the first time, the effectiveness and tolerance of MMF in EGPA remission induction. A retrospective, two-center, real-world study was conducted in patients with EGPA who received MMF in addition to prednisolone for newly diagnosed or relapsing disease between 2009 and 2019. Baseline, 3-, 6- and 12-month outcome data were extracted from electronic health records. The primary outcome was disease remission, defined as a Birmingham Vasculitis Activity Score of 0 at 6 months. Secondary outcomes included disease relapse, median prednisolone dose at 12 months and drug tolerance. In total, 15 patients (73% male, median age 57) with EGPA (11 newly diagnosed/4 relapsing) were identified. At 6 months, 67% had achieved disease remission. At 12 months, this was maintained (66.7%) and 4 patients had relapsed. All but one patient remained on MMF at study completion and all patients tolerated MMF. Our real-world data suggest that MMF is an effective and well-tolerated agent for achieving disease remission in EGPA. A future randomized controlled trial of MMF in this neglected orphan disease is now warranted.

**Supplementary Information:**

The online version contains supplementary material available at 10.1007/s00296-021-04961-w.

## Introduction

ANCA-associated vasculitis (AAV) includes granulomatosis with polyangiitis (GPA), microscopic polyangiitis (MPA) and eosinophilic granulomatosis with polyangiitis (EGPA). ANCA seropositivity among EGPA patients is approximately 40%, compared to up to 90% in those with GPA and MPA [1]. Moreover, the pathobiology of EGPA is distinct, dominated by eosinophils rather than neutrophil-based mechanisms [2]. Clinical trials in AAV have generally excluded those with EGPA. Consequently, the evidence base supporting the management of EGPA is largely inferred. The use of mycophenolate mofetil (MMF) in EGPA is one such example.

Based on expert opinion, current international clinical guidelines consider MMF as a therapeutic option for non-organ threatening EGPA when used in combination with corticosteroids [3, 4]. MMF reduces IL-5 (the key driver of eosinophil maturation and function) generation in animal models [5] in addition to mediating inhibition of T and B lymphocyte proliferation [6]. A recent meta-analysis concluded that MMF was an effective alternative remission-induction agent to cyclophosphamide in AAV. Although this analysis excluded patients with EGPA, it did identify those patients with myeloperoxidase (MPO) ANCA seropositivity, the prevailing ANCA in EGPA, to be specifically responsive [7]. Despite its wide spread use, no studies have evaluated the efficacy and tolerance of MMF for remission induction in patients with EGPA. Here, we describe a real-world experience of MMF use for this indication.

## Methods

A retrospective, observational study was performed. All patients diagnosed with EGPA between 2009 and 2019, and prescribed MMF (to a target dose of 1 g twice daily as standard) in combination with prednisolone for remission induction were identified from databases of the two largest regional vasculitis services in Scotland. These sites do not share a standardized prednisolone tapering protocol, but they generally follow a reduced dose taper, similar to that tested in the PEXIVAS trial [8], for newly diagnosed cases and a more modest starting dose (20–30 mg prednisolone) for relapsing patients. Cases were required to fulfil the 2012 Chapel Hill Consensus definition for EGPA [9].

Data were extracted from electronic health care records. Baseline characteristics (Table 1) included demographics, duration of symptoms prior to treatment, clinical manifestations, serological markers and ANCA status (Indirect Immunofluorescence (IIF) and Enzyme Linked Immunoassay (ELISA). At 0, 3, 6 and 12 months, the Birmingham Vasculitis activity score (BVAS, version3) [10] assessed disease activity, and prednisolone doses were calculated. In addition, 1-year relapse rates (defined as the recurrence of symptoms and signs suggestive of EGPA requiring augmentation of immunosuppression), drug in tolerance and infection rates were recorded.

**Table 1:** Baseline characteristics (*n* = 15)

Demography	
Sex M/F	11/4
Age, median (IQR)	57 (27–76)
Characteristics at EGPA diagnosis	
Organ involvement (%)	
Lung (including asthma)^a^	12 (80%)
ENT	9 (60%)
Neuropathy	10 (67%)
Arthropathy	7(47%)
Cutaneous	4 (27%)
Cardiac involvement	2 (13%)
Renal	1 (7%)
Median eosinophil 10^9^/l (IQR)	3.48 (0.01–21)
Positive ANCA	7 (47%)
BVAS, median (IQR)^b^	11 (4–24)

^a^Pulmonary infiltrate /nodularity was found in 9 patients(60%)

^b^Individual BVAS is provided in supplementary table

The primary outcome was complete remission (defined as BVAS of 0) at 6 months. Secondary outcomes at 12 months were (a) complete remission, (b) partial response (a reduction of BVAS ≥ 50% compared with baseline), (c) total number of disease relapses, (d) cumulative prednisolone dose, (e) drug intolerance, (f) serum C-reactive protein, (g) peripheral eosinophil count (Table 2).

**Table 2:** Primary and secondary outcomes

	Baseline	6 months	12 months
Complete remission	NA	10/15 (67%)	10/15 (67%)
Partial response	NA	3/15 (20%)	4/15 (27%)
Median cumulative prednisolone dose	NA	3.1 g (1–5.7)	4.7 g (1.9–7.9)
Total relapses	NA	2/15 (13%)	4/15 (27%)
Drug intolerance	NA	Nil reported	Nil reported
Median eosinophils count × 10^9^/l (IQR)	3.48 (0.01–21)	0.2 (0.02–1)	0.16 (0.01–0.65)
Median CRP mg/L (IQR)	13.5 (< 4–113)	< 4 (1–12)	< 4 (< 4–120)

Results were reported employing summary descriptive statistics. As this project was a service evaluation exercise, the UK Health Research Authority decision tool determined that ethical approval was not needed (NHS Grampian R&D reference 5257).

## Results

Of the 15 patients (11 males) identified, 11 had newly diagnosed EGPA and 4 had relapsing disease. At baseline (Table 1), median age (IQR) was 57(27–76) and 7/15 were MPO-ANCA positive. The remaining 8 patients were ANCA negative.

Lung, nerve and ENT were the most affected organs in our cohort. Cardiac involvement, which carries poor prognosis, was evident in 2 patients.

Amongst disease relapsers (*n* = 4), previous immunosuppression included cyclophosphamide (*n* = 3), azathioprine (*n* = 3), methotrexate (*n* = 2), and rituximab (*n* = 1).

Ten patients (66.7%) achieved the primary outcome of disease remission by 6 months, and this was maintained at 12-month follow-up. Secondary outcomes at 12 months (Table 2) revealed 4 relapses. Of these, 3 had non-organ threatening involvement (ENT or chest flares) which responded to a temporary increase in prednisolone and a longer-term increase in MMF dose (from 2 to 3 g/day), and one was a relapse of peripheral neuropathy, which required a switch from MMF to rituximab. All other patients remained on 2 g/day MMF at 1 year. No patients were intolerant of MMF. Specifically, no allergic reactions were observed nor was gastrointestinal disturbance reported. Six patients experienced infections (5 respiratory, 1 urinary), one which was considered severe. All patients with baseline peripheral blood eosinophilia, recorded a normal eosinophil count at 6 and 12 months. Median prednisolone doses were 7.5 and 5 mg/day at 6 and 12 months, while the cumulative prednisolone doses were 3.1 and 4.7 g, respectively. The prednisolone dose was increased 7 times in 7 patients.

Outcomes of newly diagnosed EGPA patients were broadly comparable to those with disease relapse, as were outcomes stratified by ANCA status where the complete remission for the positive group was achieved in 71% and 63% in the ANCA negative group (Supplementary Tables 1 and 2).

## Discussion

In this retrospective evaluation, almost all newly diagnosed and relapsing EGPA patients responded to MMF in combination with prednisolone; two-thirds successfully achieving disease remission at 6 months. Moreover, after 1 year of follow-up, all patients tolerated MMF and only one patient switched immunosuppression.

This is the first study to examine the role of MMF in EGPA. In fact, very few studies have evaluated any therapy in EGPA and only mepolizumab has been tested by a randomized controlled trial. Compared to placebo, this IL-5 blocker demonstrated superior efficacy with approximately a third of patients achieving complete remission at 1 year. Although significantly less than the 67% remission rate reported in this study, the trial of mepolizumab selectively recruited relapsing or refractory EGPA patients, by definition a more treatment resistant population [11]. In keeping with this, the remission rate for those newly diagnosed (*n* = 11) in our study was even higher 82% and 73% at 6 and 12 months, respectively) and for the relapse group (*n* = 4) the remission rate was 25% and 50% at 6 and 12 months, respectively.

Another retrospective observational study, which examined the role of rituximab in EGPA [12], comprised of patients with similar baseline levels of disease activity to our study, but again included mostly relapsing/refractory patients. This, again, likely explains the apparent lower remission rates (34% vs. 67% at 6 months and 49% vs. 67% at 12 months) and steroid sparing effects (53% vs. 67% < 10 mg prednisolone at 12 months) between RTX and MMF. The median cumulative prednisolone exposure in this study (4.7 g at 12 months) is largely similar to what was observed in an open label prospective EGPA study of methotrexate and much less than older cohorts which often did not use corticosteroid-sparing agents [13].

In comparison to other AAV subtypes, our data are remarkably similar to the outcomes of the MYCYC trial. In this randomized controlled trial of MMF versus cyclophosphamide for GPA/MPA remission induction, subjects randomized to MMF achieved complete remission rates of 67% at 6 months and relapse rates of 25% at 1 year [14]. In other cohorts, relapse rates of 50% at 21 months [13] and 44% at 24 months [15] have been reported, which compares to the 27% at 12 months observed in this study.

Key study limitations should be considered. First, this is a small sample size subject to selection bias and confounding. MMF is not the only first-line remission-induction agent used by the contributing vasculitis services and the ultimate therapeutic choice was determined by individual patient factors. Generally, MMF is used for non-organ threatening presentations, although in this cohort, *n* = 10 and *n* = 2 manifested neuropathic and cardiac involvement, respectively. Second, this study is retrospective in design. The centralization of our electronic health records helped optimize data capture but the possibility of measurement error when computing granular outcomes, such as cumulative prednisolone dose and measuring BVAS score, cannot be excluded. Third, without a placebo comparison group, it is not possible to attribute the observed outcome improvements to MMF alone. Contextualization with historical cohorts is helpful but direct comparisons are imperfect since many of the outcomes are not identically defined. A randomized controlled trial is essential to determine true efficacy. Fourth, 1-year follow-up of subjects is adequate to evaluate remission induction but insufficient to evaluate remission maintenance.

In summary, MMF appears to be well tolerated and effective for inducing disease remission among patients with newly diagnosed or relapsing EGPA. Despite differences with GPA/MPA, these data are the first to support existing expert-based guidelines which recommend MMF as a useful remission induction option across the spectrum of AAV. Although reassuring, this is a small observational study vulnerable to confounding. Well-designed randomized controlled trials are imperative to full ascertain the role of this drug in this frequently neglected burdensome condition.

## Supplementary Information

Below is the link to the electronic supplementary material.

Supplementary file1 (DOCX 15 KB)
