# Supplementary material for: A real-world assessment of mycophenolate mofetil for remission induction in eosinophilic granulomatosis with polyangiitis
Source: Rheumatol Int. 2021 Aug 4;41(10):1811–4. doi: 10.1007/s00296-021-04961-w (PMC8390413; doi:10.1007/s00296-021-04961-w)
Supplement: Supplementary file 1 — Supplementary file1 (DOCX 15 KB) [file 296_2021_4961_MOESM1_ESM.docx]

**Supplementary table 1: 12-month outcomes stratified according to new diagnosis/relapse**

|  | Baseline | | 3 months | | 6 months | | 12 months | |
| --- | --- | --- | --- | --- | --- | --- | --- | --- |
| MMF Induction=11  Relapse=4 | Newly  diagnosed | Relapse | Newly  diagnosed | Relapse | Newly diagnosed | Relapse | Newly  diagnosed | Relapse |
| Median BVAS(IQR) | 12(7-24) | 8.5(4-10) | 3(0-11) | 0(0) | 0(0-7) | 2.5  (0-10) | 0  (0-2) | 1.5  (0-8) |
| Remission rate | NA | NA | 18%  (2/11) | 100% | 82%  (9/11) | 25%  (2/4) | 73%  8/11 | 50%  2/4 |
| Median Prednisolone dose mg/day  (IQR) | 40  (20-60) | 22.5  (15-30) | 15  (10-20) | 8.75  (5-10) | 8.75  (5-15) | 7.5  (5-15) | 5  (5-30) | 8.75  (5-20) |
| Median  Cumulativepredinslonedosemg(IQR) | NA | NA | 2506mg  (1460-4140) | 1316.5mg  (565-2165) | 3587.5  2160-5700) | 2045  (1020-3127.5) | 5192.5  (3105-7950) | 3741.5  (1930-5347.5) |
|  |  |  |  |  |  |  |  |  |
| Median VDI | NA | 1(0-2) | NA | Not done | 1(0-3) | 1(0-2) | 1(0-3) | 2(0-2) |
| Eosinophils  Median(IQR) | 5.3(0.07-21) | 0.165  (0.01-0.3) | Not done | Not done | 0.21(0.02-0.8) | 0.2(0.1-0.3) | 0.15(0.01-0.65) | 0.12(0.1-0.4) |

**Supplementary table 2: 12-month outcomes stratified by baseline ANCA status**

| **ANCA** | **Complete**  **Remission** | **Partial response** | **Total relapses(%)** | **Median Cumulative preddoses(IQR)** | **Eosinophils**  **x10^9/l (IQR)** | **CRP**  **mg/L**  **(IQR)** |
| --- | --- | --- | --- | --- | --- | --- |
| **Positive**  **(n=7)** | 5(71%) | 2(29%) | 2(29%) | 5.6 g(3.1-7.9) | 0.15(0.04-0.5) | <4  (4-120) |
| **Negative**  **(N=8)** | 5(63%) | 2(25%) | 2(25%) | 4.45g(1.9-5.6) | 0.2(001-0.65) | <4  (1-5) |

**Supplementary table 3: Individual BVAS and prednisolone dose**

| **Patients** | **BVAS** | | | | **Prednisolone dose** | | | |
| --- | --- | --- | --- | --- | --- | --- | --- | --- |
|  | **At baseline** | **At**  **3 months** | **At**  **6 months** | **At**  **12 months** | **At baseline** | **At**  **3 months** | **At**  **6 months** | **At**  **12 months** |
| 1 | 15 | 0 | 0 | 2 | 60 | 15 | 10 | 10 |
| 2 | 12 | 0 | 0 | 0 | 40 | 15 | 7.5 | 5 |
| 3 | 11 | 6 | 0 | 0 | 60 | 15 | 10 | 5 |
| 4 | 24 | 11 | 0 | 0 | 25 | 10 | 12.5 | 20 |
| 5 | 9 | 1 | 2 | 0 | 40 | 15 | 10 | 5 |
| 6 | 16 | 3 | 0 | 2 | 20 | 15 | 10 | 30 |
| 7 | 11 | 3 | 7 | 0 | 60 | 20 | 15 | 7.5 |
| 8 | 8 | 0 | 3 | 3 | 30 | 10 | 7.5 | 7.5 |
| 9 | 10 | 0 | 10 | 0 | 20 | 10 | 15 | 10 |
| 10 | 4 | 0 | 0 | 0 | 15 | 5 | 5 | 5 |
| 11 | 9 | 0 | 2 | 8 | 25 | 7.5 | 7.5 | 20 |
| 12 | 20 | 4 | 0 | 0 | 60 | 12.5 | 7.5 | 5 |
| 13 | 12 | 3 | 0 | 0 | 35 | 10 | 7.5 | 5 |
| 14 | 7 | 4 | 0 | 0 | 60 | 15 | 7.5 | 5 |
| 15 | 14 | 4 | 0 | 2 | 20 | 10 | 5 | 5 |
